# Effects of using the nursing care model for the prevention of adverse events in critically ill patients undergone arterial line insertion

**DOI:** 10.1017/ash.2025.94

**Published:** 2025-09-03

**Authors:** Narin Pipattanawarrakhun, Piyaporn Teerasin

**Affiliations:** Surgical Intensive Care Unit, Division of Nursing, NakhonPathom Hospital, Nakhon Pathom, Thailand

## Abstract

**Objectives:** The most common adverse events (AEs) of an arterial line (A-line) insertion included inflammation, infection, bloodstream infection (BSI), disconnection, and occlusion of the device, etc., the purposes of this study were to compare the nursing practice of registered nurses before and after using the nursing care model for the prevention of AEs and to compare the incidence of AEs. **Methods:** This quasi-experimental study was conducted among registered nurses (n = 14) and critically ill patients who received A-line insertion (n = 40), which were divided into either a control group (n = 20) or an experimental group (n = 20). The tools used included 1) personal data and clinical data recording form, 2) AEs recording form, 3) the nursing care model for prevention AEs in patients who received A-line insertion comprising five methods “ABCD’S of care nursing care model”; 1) assessment of the AEs, 2) blood sampling, 3) cleansing and closed A-line site with the use innovation “Tegaderm with Window for A-line” and circuit care, 4) daily review, and 5) standard of care and 4) a nursing practice behavior assessment form. Data analysis involved descriptive statistics, t-tests, and chi-square tests. **Results:** The average nursing practice behavior scores increased from 2.57 points (SD = 0.51) to 4.5 points (SD = 0.52), indicating a substantial improvement. Moreover, the incidence of AEs decreased from 45% to 5%, a remarkable reduction. These findings underscore the effectiveness of the nursing care model in preventing AEs in critically ill patients. **Conclusion:** Based on the “ABCD’S of care”, the nursing care model has proven effective in reducing the incidence of AEs in critically ill patients. This finding enhances our understanding of nursing practices and provides a practical solution for healthcare professionals. It is, therefore, crucial to disseminate and implement these guidelines to ensure sustainable nursing practices.

